# Burst dynamics enable contrast coding via synchrony

**DOI:** 10.1186/1471-2202-12-S1-P34

**Published:** 2011-07-18

**Authors:** Oscar Ávila Åkerberg, Maurice J Chacron

**Affiliations:** 1 Department of Physics, McGill University, Montreal, Quebec, Canada, H3A2T8; 2 Department of Physiology, McGill University, Montreal, Quebec, Canada, H3G1Y6

## 

Neurons in various sensory systems respond to stimuli with a variety of action potential patterns including isolated action potentials and bursts (i.e. high frequency groups of action potentials). There is accumulating evidence that, instead of being just a collection of action potentials, bursts are instead a specific pattern that signals stimulus attributes that are distinct than those signaled by isolated action potentials [1]. Specifically, it has been proposed that synchronous bursts can signal the occurrence of certain stimuli [2] and encode higher order stimulus attributes such as contrast [3]. Here we test experimentally whether synchronous activity in a population of neurons can encode a time varying stimulus contrast. To do so, we performed intracellular recordings of the activity of electrosensory pyramidal cells in-vitro. These cells have been well characterized and previous studies have shown that they tonically fire isolated action potentials under control conditions but that application of the neuromodulator serotonin (5-HT) can cause them to transition into a burst firing mode [4]. As such, we tested the information transmission capabilities of these cells about stimulus contrast in both modes. To do so, we provided the cells with a time-varying stimulus and investigated how the number of synchronous spikes correlates with stimulus contrast. We found that synchrony had a larger dependence on stimulus contrast in pyramidal cells after 5-HT injection than under control conditions. These experimental results agree with the predictions of the model presented in [2]. Furthermore, we developed a mathematical theory that supports our experimental results and proposes that the change in coding properties between bursting and non-bursting neurons is solely attributed to the higher baseline response variability as quantified by the coefficient of variation (CV) present in bursting neurons. Our experimental results therefore show that bursting neurons are more apt at coding stimulus contrast through synchronous firing than tonic ones. Moreover, our theoretical results suggest that this occurs because burst dynamics introduce variability in the responses of single neurons. The latter is supported by the fact that bursting neurons with the highest CVs showed the greatest dependency on stimulus contrast in their synchronous activities. Our results thus provide the first experimental evidence that bursting neurons can code for stimulus contrast via their synchronous activity and provide a novel function for burst dynamics.
